# LC3-associated phagocytosis is impaired in monocyte-derived macrophages from systemic sclerosis patients

**DOI:** 10.1080/27694127.2026.2730912

**Published:** 2026-10-06

**Authors:** Quentin Frenger, Julie Lucas, Arthur Petitdemange, Luisa Path, Nadège Wadier, Sabine Depauw, Stéphane Giorgiutti, Yazhuo Gong, Hélène Merlin, Aurore Meyer, Mathilde Herber, Léa Jaquel, Philippe Mertz, Renaud Felten, Emmanuel Chatelus, Yannick Dieudonne, Aurélien Guffroy, Laurent Arnaud, Vincent Poindron, Jacques-Eric Gottenberg, Jean Sibilia, Anne-Sophie Korganow, Thierry Martin, Frédéric Gros

**Affiliations:** a Faculty of Life Sciences, University of Strasbourg, Strasbourg, France; b Inserm UMR_S 1109 - Immuno-Rhumatologie moléculaire, University of Strasbourg, Strasbourg, France; c Department of Clinical Immunology and Internal Medicine, Hôpitaux Universitaires de Strasbourg, Strasbourg, France; d Faculty of Medicine, University of Strasbourg, Strasbourg, France; e National Reference Center for Systemic Autoimmune Diseases (CNR RESO), Tertiary Center for Primary Immunodeficiency, Strasbourg, France; f CNRS UPR3572 - Immunology, Immunopathology and Therapeutic Chemistry, Institute of Molecular and Cellular Biology, University of Strasbourg, Strasbourg, France; g Department of Rheumatology, National Reference Center for Systemic Autoimmune Diseases (CNR RESO), Hôpitaux Universitaires de Strasbourg, Strasbourg, France

**Keywords:** Autophagy, conjugation of ATG8 to single membranes (CASM), LC3-associated phagocytosis, autoimmunity, systemic sclerosis, efferocytosis, macrophages

## Abstract

Autophagy-related (ATG) proteins orchestrate canonical autophagy and related pathways, including LC3-associated phagocytosis (LAP). Autophagy and LAP play essential roles in immune homeostasis and regulation. Systemic sclerosis (SSc) is an autoimmune disease of unknown etiology characterized by progressive fibrosis affecting the skin and multiple internal organs, together with profound immune dysregulation. *ATG* alleles have been proposed as genetic susceptibility factors for SSc, suggesting that defects in autophagy-related pathways may contribute to the disease pathogenesis. We investigated whether canonical autophagy and/or LAP are altered in immune cells from patients with SSc. Autophagy was assessed by measuring LC3-II levels in circulating cells from SSc patients and controls, and autophagic flux was evaluated by flow cytometry following lysosomal inhibition. We then generated monocyte-derived macrophages and performed phagocytosis assays to assess the LAP activity. We evaluated the phagocytic capacity using IgG-coated beads that trigger LAP through Fcγ receptor engagement and measured the proportion of LC3-positive phagosomes (LAPosomes). No major defects in canonical autophagy were found in lymphocytes and monocytes isolated from the peripheral blood of patients with SSc. In contrast, monocyte-derived M0 macrophages from patients with SSc exhibited impaired LC3-associated phagocytosis. Impaired LAP in macrophages may contribute to the defective efferocytosis observed in SSc, thereby promoting persistent inflammation and fibrosis. Our findings identify LAP as a previously unrecognized pathway altered in SSc and suggest that therapeutic strategies aimed at restoring LAP activity may represent a novel approach for disease treatment.

## KEY MESSAGES

- Polymorphisms in autophagy-related genes were associated with the development of SSc.
- A defect in LC3-associated phagocytosis (LAP) has been found in monocyte-derived macrophages from patients with SSc.
- This LAP deregulation could affect efferocytosis and thus contributes to systemic inflammation and fibrosis.

## Introduction

Systemic sclerosis (SSc) is an autoimmune disease characterized by progressive fibrosis involving the skin and internal organs [1]. Fibroblast dysregulation is thought to result, at least in part, from chronic inflammation driven by both innate and adaptive immune cells [2]. Alterations in the phagocytic compartment have also been implicated in disease pathogenesis, as illustrated by the imbalance between classically activated (M1) and alternatively activated (M2) macrophages reported in several studies [3–5]. More recently, impaired efferocytosis has emerged as another hallmark of phagocyte dysfunction in SSc and may be linked to abnormal macrophage polarization [6]. Consistent with this hypothesis, exposure to crystalline silica – an established environmental risk factor for SSc – has also been shown to impair efferocytosis [7].

ATG (autophagy-related) proteins constitute the core machinery responsible for macroautophagy (hereafter referred to as autophagy). In adaptive immune cells, autophagy is essential for the survival of long-lived cells, including memory lymphocytes and plasma cells [8]. In phagocytes, autophagy contributes to the elimination of intracellular pathogens through the formation of double-membrane autophagosomes. It also limits inflammation by restraining inflammasome activation and type I interferon (IFN-I) production, while promoting the presentation of cytosolic antigens [9]. In addition to canonical autophagy, some ATG proteins can contribute to the conjugation of LC3 (light chain 3; MAP1LC3, the mammalian orthologue of yeast Atg8) to single-membrane intracellular vesicles through a group of processes collectively referred to as CASM (conjugation of ATG8 proteins to single membranes) [10,11]. LC3-associated phagocytosis (LAP), the best-characterized form of CASM, plays a specialized role in professional phagocytes [12]. Although its full physiological functions remain incompletely understood, LAP has been implicated in efferocytosis, antigen presentation, intracellular vesicle trafficking, and the regulation of inflammatory responses [13]. Notably, during efferocytosis, LAP limits the production of type I IFNs and interleukin-1β [14] during efferocytosis. Collectively, these observations suggest that ATG-dependent pathways may play a central role in the pathogenesis of chronic inflammatory diseases.

Polymorphisms in ATG5 and other autophagy-related genes have been associated with susceptibility to several inflammatory diseases, including systemic sclerosis [15]. However, the contribution of ATG-dependent pathways to SSc pathogenesis remains poorly understood. To date, few studies have investigated the role of ATG proteins in SSc, and none has specifically examined autophagy-related pathways in immune cells from patients, particularly in professional phagocytes. Because dysregulation of canonical autophagy and/or LAP has been implicated in the pathogenesis of several autoimmune diseases, including Crohn’s disease, systemic lupus erythematosus (SLE), multiple sclerosis, Sjögren’s syndrome, and rheumatoid arthritis [16], we hypothesized that similar alterations may also contribute to SSc.

In the present study, we therefore investigated whether defects in canonical autophagy are detectable in circulating immune cells from patients with SSc and could promote the persistence of autoreactive immune cells. As no major alterations in canonical autophagy were identified, we next examined whether LC3-associated phagocytosis (LAP) is impaired in monocyte-derived macrophages, where it could contribute to the abnormal macrophage phenotype and defective efferocytosis associated with SSc.

## Patients, material, and methods

### Patients and healthy donors

The patients included in this study had diffuse cutaneous SSc (dcSSc) and were aged >18 years. They were recruited from the Department of Clinical Immunology and Rheumatology at Strasbourg University Hospital. The control samples were purchased from the “Établissement Français du Sang” (EFS) in Strasbourg. The patients fulfilled the American College of Rheumatology (ACR)/European Alliance of Associations for Rheumatology (EULAR) classification criteria for their respective diseases (Table 1). Written informed consent was obtained from all participants regarding the study protocol and the objectives. This study was performed under the authorization of RESO (https://maladie-autoimmune.fr), a reference center for autoimmune diseases hosted at Strasbourg University Hospital. All methods were performed in accordance with relevant guidelines and regulations. Patients and healthy individuals were matched for age and sex, and blood samples were collected in EDTA tubes and processed with minimal delay.

**Table 1.** Patient information.

Age	Sex	Age of first sign	Disease duration	Clinical manifestations	ACR/EULAR score	Treatments	Auto-antibodies
49	M	48	5 months	Lungs	10	None	SCL-70
51	M	48	2 years and 10 months	Skin, lungs, kidneys, digestive	21	Cyclophosphamide	SCL-70
78	F	73	5 years	Skin	23	MMF	RNA-Pol-III
62	F	51	11 years and 1 month	Skin, heart, lungs	26	Glucocorticoids (12.5 mg/day)	Centromere
64	F	51	13 years and 4 months	Skin, heart, digestive	24	N-C	Centromere
62	F	61	7 months	Skin, heart, lungs	23	MMF, glucocorticoids (12.5 mg/day)	SCL-70
64	F	58	6 years	Skin, heart, kidneys	16	MMF, glucocorticoids (12.5 mg/day)	nuclear
51	F	50	1 year and 2 months	Skin	19	None	SCL-70
44	F	41	3 years and 2 months	Skin, lungs	21	None	SCL-70
38	M	37	11 months	Articular	10	None	SCL-70
40	M	37	2 years and 11 months	Articular	10	Methotrexate	SCL-70, RNA-Pol-III
81	F	78	2 years and 6 months	Skin, heart	25	Glucocorticoids (12.5 mg/day)	RNA-Pol-III
58	M	55	2 years and 11 months	Skin	19	MMF	SCL-70
59	M	55	4 years and 11 months	Skin, lungs	20	MMF	SCL-70
66	M	65	1 year and 1 month	Skin, heart, lungs, kidneys, digestive	27	Aspirin	SCL-70
29	F	27	1 year and 8 months	Skin, heart, lungs, digestive	21	None	SCL-70
44	M	44	3 months	Skin, digestive	14	None	SCL-70
29	M	23	6 years and 5 months	Skin	12	None	SCL-70
35	F	34	8 months	Skin, lungs, digestive	25	MMF	SCL-70
46	M	45	1 year and 8 months	Skin, lungs, digestive	27	MMF	SCL-70, centromere
36	F	32	3 years and 3 months	Skin, lungs	16	MMF	SCL-70
18	M	17	1 year and 5 months	Skin, lungs, digestive, articular	26	MMF, glucocorticoids (12.5 mg/day)	SCL-70
25	F	23	1 year and 8 months	Skin	20	None	SCL-70
55	M	55	7 months	Skin	18	MMF, glucocorticoids (12.5 mg/day)	Nuclear
47	F	45	2 years and 7 months	Skin	19	None	SCL-70
25	F	20	4 years and 10 months	Skin, lungs, digestive	26	MMF	SCL-70
41	F	32	8 years and 2 months	Skin, lungs, digestive	29	None	SCL-70
47	F	34	12 years and 6 months	Skin, lungs, digestive	32	None	SCL-70
60	F	59	1 year and 5 months	Skin, lungs	18	MMF	SCL-70
58	F	Unknown	2 years and 5 months	Skin, lungs, digestive	21	MMF	SCL-70
Age	Sex	Age of first sign		Clinical manifestations	SLEDAI score	Treatments	Auto-antibodies
43	F	25	Unknown	Skin, articular, pericarditis	0	Hydroxychloroquine	dsDNA, antinucleosome, anti-SSA
42	F	36	6 years	Skin	2	Glucocorticoids (5 mg/day)	dsDNA, anti-SSA
47	F	43	4 years	Skin, articular	8	NSAID	dsDNA, anti-SSA
54	F	43	11 years	Skin, articular	2	Hydroxychloroquine, glucocorticoids (12.5 mg/day)	dsDNA

Age, sex, age at first signs, disease duration, clinical manifestations autoantibody production, American College of Rheumatology/European Alliance of Associations for Rheumatology (ACR/EULAR) score (for systemic sclerosis patients), Systemic Lupus Erythematosus Disease Activity Index (SLEDAI) score (for systemic lupus erythematosus patients). Articular involvement was defined as inflammatory arthralgia or scleroderma-associated arthritis. ANA stands for “anti-nuclear” when no more information is known about its specificity. M, male; F, female; MMF, mycophenolate mofetil; SCL-70, topoisomerase I; RNA-Pol-III, RNA polymerase III; NSAID: nonsteroidal anti-inflammatory drugs; dsDNA, double-stranded deoxyribonucleic acid; anti-SSA, anti-Sjögren‘s syndrome-related antigen A autoantibodies.

### Peripheral blood mononuclear cells (PBMC) isolation from blood

Blood was diluted 1:1 with preheated PBS (phosphate-buffered saline) and layered on top of a Ficoll layer (blood/Ficoll ratio: 2:1). Samples were centrifuged at 1200 × *g* for 20 min with acceleration and brakes set to a minimum. PBMCs were harvested and washed with phosphate-buffered saline (PBS).

Platelets were removed by two additional centrifugation steps at 120 × *g* for 10 min, with brake and acceleration set to low, and the cells were then counted.

### T-cell isolation and *in-vitro* stimulation

T cells were isolated from freshly harvested PBMCs using a human Pan T-cell isolation kit according to the manufacturer’s instructions. Cells (2 × 10^5^) were seeded per well in 96-well plates and stimulated overnight with 50 ng/mL phorbol 12-myristate 13-acetate (PMA) and 1 µM ionomycin or left unstimulated.

### *In-vitro* monocyte-derived macrophage differentiation and phenotyping

Monocytes were purified from freshly isolated PBMCs by CD14-positive magnetic sorting following the manufacturer’s instructions. Monocytes were then seeded in multiwell plates at a density of 1 × 10^5^ cells/cm^2^ and incubated for 3 days at 37°C and 5% CO_2_ in the presence of 50 ng/mL macrophage colony-stimulating factor (M-CSF) for unpolarized macrophages (M0) and M2 differentiation, and 10 ng/mL granulocyte-macrophage colony-stimulating factor (GM-CSF) for M1 differentiation. On day 3, cytokines were renewed. On day 6, 10 ng/mL lipopolysaccharide (LPS) and 50 ng/mL IFN-γ were added for M1 differentiation, whereas 20 ng/mL interleukin-4 (IL-4) was added for M2 differentiation. On day 7, macrophages were harvested with Accutase, and 1 × 10^5^ cells from M0, M1, or M2 differentiation conditions were stained for extracellular markers (anti-CD163-AF647, anti-CD206-PerCP-eFluor710, anti-CD282-AF488, anti-CD14-BV510, anti-HLA-DR-BV711, anti-CD16-APC/Cyanine7, anti-CD80-PE/Cyanine7, anti-CD86-PE/CF594, and anti-CD369-PE) for phenotypic analysis by flow cytometry. Flow cytometric data were acquired using an Attune NxT flow cytometer (Thermo Fisher Scientific) and analyzed using FlowJo version 10 (LLC).

### Quantification of LC3-II accumulation by flow cytometry

A total of 1 × 10^5^ PBMCs, M0 monocyte-derived macrophages, or purified T cells were seeded per well in 96-well plates. Cells were treated with Autophagy Reagent A (bafilomycin A1, BafA1) or left unstimulated for 2 h at 37°C in 5% CO_2_ in complete medium (RPMI 1640 supplemented with 10% (v/v) heat-inactivated fetal bovine serum and 50 µg/mL gentamicin). Cells were stained with anti-CD3-APC-AF750, anti-CD19-APC, and anti-CD14-PerCP-Cy5.5 and then permeabilized with 0.05% (m/v) saponin. Soluble LC3-I was removed by washing cells at 500 × *g* for 5 min, and cells were then stained with anti-LC3-FITC antibody. Acquisition was performed in assay buffer using a Gallios flow cytometer (Beckman Coulter), and data were analyzed using FlowJo version 10 (LLC). Autophagic flux was calculated by dividing the LC3-FITC mean fluorescence intensity (MFI) of the BafA1-treated condition by the MFI of the untreated condition.

### Phagocytosis assays and anti-LC3B immunofluorescence

A total of 100 µg of human immunoglobulin G (IgG, Sigma-Aldrich, 14506) or bovine serum albumin (BSA) was covalently linked to 3 µm Polybead® Carboxylate Microspheres with PolyLink Protein Coupling Kit following the manufacturer’s instructions. A total of 2 × 10^5^ macrophages were seeded per well on eight-chamber slides and left to attach overnight. A total of 8 × 10^5^ beads were incubated with macrophages for 45 min at 37°C with 5% CO_2_ in complete medium. The cells were then washed twice with ice-cold PBS to remove the free beads. The cells were fixed with 4% (w/v) paraformaldehyde diluted in PBS for 10 min at room temperature and washed three times with PBS. The cells were then permeabilized by incubation for 30 min with 0.05% (v/v) Triton X-100 diluted in PBS containing 2% BSA (w/v). Blocking was performed by incubating with PBS-2% BSA (w/v) for 1 h. The primary anti-LC3B antibody, diluted in PBS containing 2% BSA, was then incubated overnight at 4°C. The cells were washed five times with PBS and incubated for 1 h with goat antirabbit IgG Alexa Fluor™ Plus 647 secondary antibodies diluted in PBS containing 2% BSA. Cells were washed five times for 5 min with PBS and mounted using ProLong™ Glass Antifade Mountant with NucBlue™. After overnight curing, the slides were randomly imaged with a Zeiss LSM 800 AiryScan microscope with a 40×/1,4 (DIC) NA objective. Optical z-sections of 1 µm were acquired. To prevent operator bias and ensure reproducibility, image analysis was performed independently by two blinded observers. Phagosomes and LAPosomes were quantified using Fiji (https://imagej.net/). To standardize the identification of LAPosomes across samples, a semi-automated thresholding approach was applied. Specifically, an intensity threshold corresponding to the top 10% of the maximum LC3 fluorescence signal was established. LAPosomes were strictly defined as phagosomes surrounded by an LC3 fluorescent ring exceeding this predefined threshold.

### Statistics

Data are reported as means ± standard deviation (SD) and were analyzed using a two-tailed Wilcoxon test, two-way ANOVA with Tukey’s multiple comparisons test, or Kruskal–Wallis test with Dunn’s multiple comparison test as appropriate. Statistical analyses were performed using GraphPad Prism 8 (GraphPad Software) or with the Rstatix package implemented in R 4.2.2. All statistical analyses were considered statistically significant at *p* < 0.05.

### Antibody and reagents

Antibody and reagents are described in Table 2.

**Table 2.** Reagent list and references.

Reagent	Concentration or dilution	Reference
8 Chamber slides		Milipore, PEZGS0816
PBS		Sigma-Aldrich, D8537
Ficoll Paque PLUS		Sigma-Aldrich, 17-1440-03
RPMI 1640		Gibco™, 21875034
Fetal bovine serum	10% (v/v)	Gibco™, A3160802
Gentamycin	50 µg/mL	Gibco™, 15750037
Accutase	–	Gibco™, A1110501
Pan T cell Isolation Kit, human	–	Miltenyi Biotec, 130-096-535
CD14 MicroBeads, human	20 µL/10^7^ cells	Miltenyi Biotec, 130-050-201
Autophagy Reagent A	1/1000	Luminex®, FCCH10017
Phorbol myristate acetate	50 ng/mL	Invivogen, tlrl-pma
Ionomycin	1 µM	Invivogen, inh-ion-3
M-CSF	50 ng/mL	R&D, 216-MC
GM-CSF	10 ng/mL	Gift from F. Oberling (CHUS)
Lipopolysaccharide	10 ng/mL	Sigma-Aldrich, 297-473-0
IFN-γ	50 ng/mL	Peprotech, 200-04
IL-4	20 ng/mL	Peprotech, 200-04
Saponin	0.05% (w/v)	Sigma-Aldrich, S4521
Triton X-100	0.05% (v/v)	Euromedex, 2000-C
Paraformaldehyde	4% (w/v)	EMS, 15710
ProLong™ Glass		Invitrogen, P36985
Human Immunoglobulin type G	–	Sigma-Aldrich, 14506
Bovine Serum Albumin	–	Euromedex, 04-100-812-E
3 µm Carboxylate Microspheres	–	Polysciences, 09850-5
Protein Coupling Kit	–	Polysciences, 24350-1
Anti CD3-APC-AF750 (UCHT1)	FC: 1/100	Beckman Coulter, A94680
Anti CD19-APC (HIB19)	FC: 1/100	BD Bioscience, 555415
Anti CD14-PerCP-Cy5.5 (M5E2)	FC: 1/100	BD Bioscience, 550787
Anti LC3-FITC (4E12)	FC: 1/20	Luminex®, FCCH10017
Anti LC3 (polyclonal)	IF: 1/200	Abcam, ab48394
Anti CD163-AF647 (MAC-2–158)	FC: 1/400	Invitrogen, 51-1637-42
Anti CD282-AF488 (TL2.1)	FC: 1/100	Luminex®, FCCH10017
Anti CD206-PerCP-eFluor 710 (19.2)	FC: 1/100	Invitrogen, 46-2069-42
Anti CD14-BV510 (MφP-9)	FC: 1/200	BD Biosciences, 563079
Anti HLA-DR-BV711 (G46-6)	FC: 1/500	BD Biosciences, 563696
Anti CD16-APC-Cy7 (3G8)	FC: 1/500	BD Biosciences, 302018
Anti CD80 -PE-Cy7 (L307,4)	FC : 1/400	BD Biosciences, 561135
Anti CD86-PE-CF594 (2331)	FC : 1/500	BD Horizon, 562390
Anti CD369-PE (15E2)	FC : 1/100	Invitrogen, 12-9856-42
Anti Rabbit-IgG-AF + 647 (polyclonal)	IF: 1/500	Invitrogen, P36985

## Results

### Evaluation of autophagic activity in PBMC from SSc patients

Autophagy contributes to autoreactive lymphocyte survival. As we and others have previously described autophagy dysregulation in T and B lymphocytes from systemic autoimmune diseases, we investigated the levels of LC3B-II by flow cytometry in PBMC from SSc patients and age- and sex-matched healthy individuals. All patients fulfilled the 2013 ACR/EULAR classification criteria [17] (Table 1). Briefly, cells were treated with saponin without prior fixation to eliminate soluble cytosolic LC3-I and to specifically stain membrane-associated LC3-II. This staining method allows the assessment of LC3-II levels in B and T lymphocytes, as well as monocytes in each sample (Figure 1(A,B)). We did not detect significant differences in LC3-II levels at steady state between healthy donors (HD) and SSc patients in T cells, B cells, or monocytes (Figure 1(C)). LC3-II can be used as a marker for autophagosomes but is also associated with other intracellular vesicles during CASM. To focus on autophagic activity, we assessed autophagic flux by treating the cells with BafA1, which blocks lysosomal acidification. As LC3-II is continuously recycled during the final steps of autophagy, this treatment leads to the accumulation of LC3-II positive autophagosomes. Although autophagic flux was detectable in T cells and monocytes, no difference in LC3-II accumulation was observed between the HD and SSc samples (Figure 1(D)). However, we observed a slightly reproducible flux increase in SSc B cells compared to HD (observed in five out of the seven patients tested). Flux values are, however, low compared to those of monocytes with high constitutive autophagic flux (one to two versus one to five), questioning the relevance of this increase in autophagic activity. As it was previously described that T-cell activation leads to the accumulation of LC3-II, we examined PMA-ionomycin-stimulated cells to determine whether differences could be observed under-activated conditions. We found a trend toward a higher flux in control T cells than in nonstimulated cells (1–2 compared with 1.5–3.5). Consistent with the results obtained in fresh PBMCs, unstimulated T cells showed no difference in LC3-II expression levels or flux between HD and SSc samples. We report, however, a slight increase in LC3-II levels at the basal level and a decrease after BafA1 treatment. This could reflect a mild alteration in the autophagic process at the degradation step in SSc T cells, as suggested by flux calculation (Figure 1(D)).

**Figure 1.**
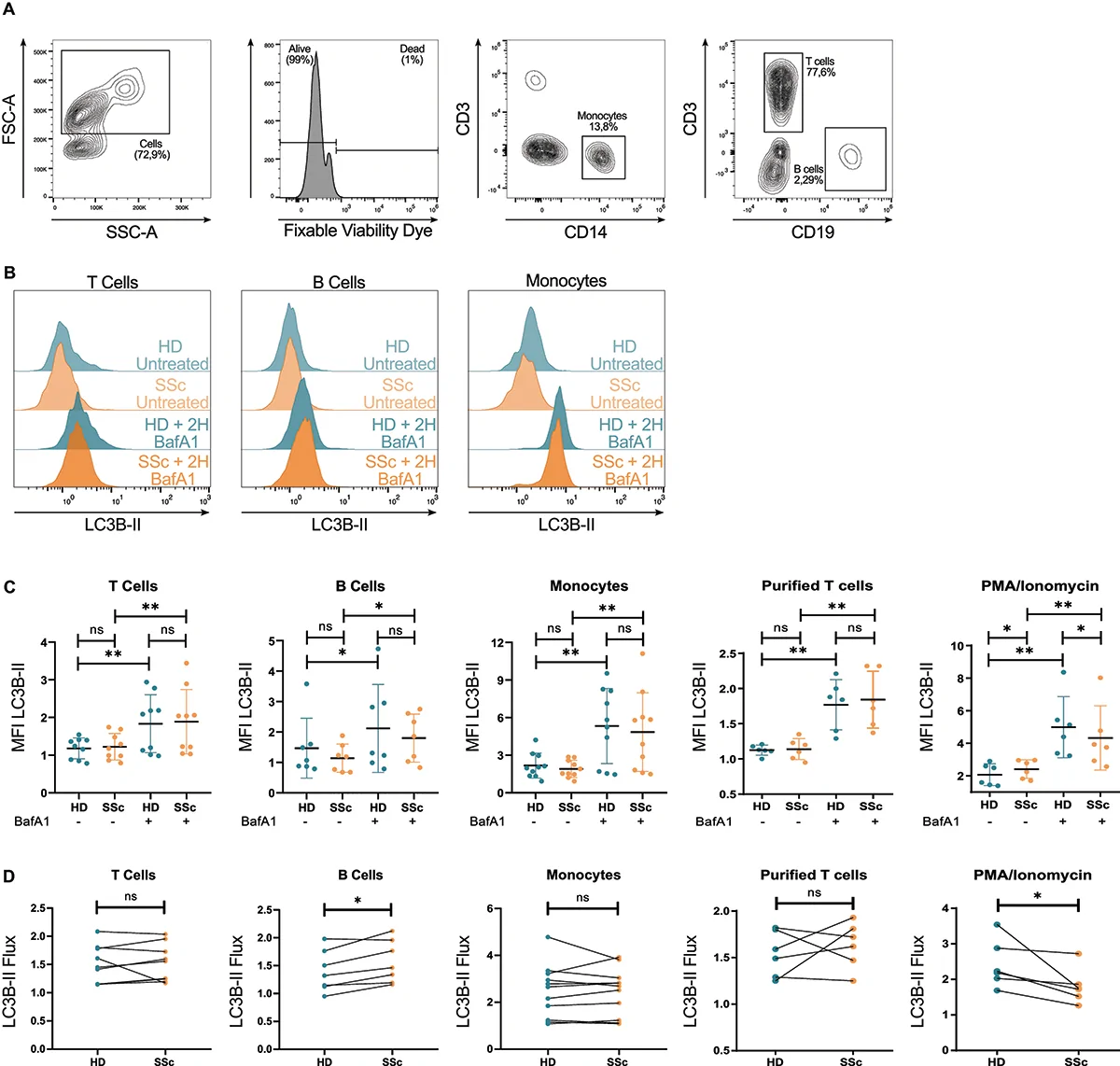
Autophagic flux is preserved in PBMC subsets from SSc patients compared to healthy controls. (A) Gating strategy used to identify B cells (CD19+, CD3−, CD14−), T cells (CD3+, CD19−, CD14−), and monocytes (CD14+, CD19−, CD3−) from the peripheral blood of healthy donors (HD) and patients with systemic sclerosis (SSc). (B) Representative flow cytometry histograms of LC3-II mean fluorescence intensity (MFI) in viable T cells, B cells, and monocytes following Bafilomycin A1 treatment. (C) Quantification of LC3-II MFI after Bafilomycin A1 treatment. (D) Calculated autophagic flux in resting T cells (*n* = 9), B cells (*n* = 7), and monocytes (*n* = 10), as well as in purified T cells (*n* = 6) left untreated or stimulated with PMA/ionomycin. Statistical analyses were performed using a two-tailed Wilcoxon test.

### Differentiation and phenotypes of polarized monocyte-derived macrophages from SSc and SLE patients

We investigated autophagy in monocyte-derived macrophages. To this end, we differentiated macrophages from purified circulating CD14^+^ monocytes. As autophagy could be regulated differently according to macrophage polarization, we wanted to determine which skewing condition resulted in the most comparable phenotypic profile between the control and SSc macrophages. We therefore differentiated macrophages from purified circulating monocytes under M0, M1, or M2 skewing conditions (Figure 2(A)). For comparison, we also included samples from another systemic autoimmune disease, SLE. First, we ensured that cell viability was comparable across the different polarizing conditions and pathologies (Figure S1). We found preferential expression of the expected markers in the corresponding polarization settings for HD, as well as SSc and SLE samples (HLA-DR, CD80, and CD86 for M1 cells; CD163 and CD16 for M0 and M2 cells; and Dectin 1 for M2 cells). CD206 levels were low in all conditions and did not allow reliable discrimination of M2 cells. This may be due to the use of GM-CSF for M1 cell differentiation, which is known to induce CD206 expression [18]. However, we are confident in the consistency of the M2 phenotype based on the statistically increased expression of Dectin-1 reported in several studies [19]. Interestingly, we found a tendency for decreased HLA-DR and CD86 expression in the SSc and SLE M1 samples (Figure 2(B)). We also found that, under M1 skewing conditions a high proportion of cells from SSc and SLE patients co-expressed CD163 and CD80, indicating a potential bias to an intermediate M1/M2 phenotype (Figure 2(C,D)). This finding is consistent with previous reports describing a propensity toward M2-like macrophage differentiation or mixed M1/M2 phenotypes in SSc tissues [20]. We found no major difference in M0- or M2-associated marker expression between SSc, SLE patients, and HD macrophages. We then assessed the LC3-II flux in M0 macrophages from four patients with SSc. As for undifferentiated monocytes, we found no evidence of altered autophagy in these cells (Figure 2(E)).

**Figure 2.**
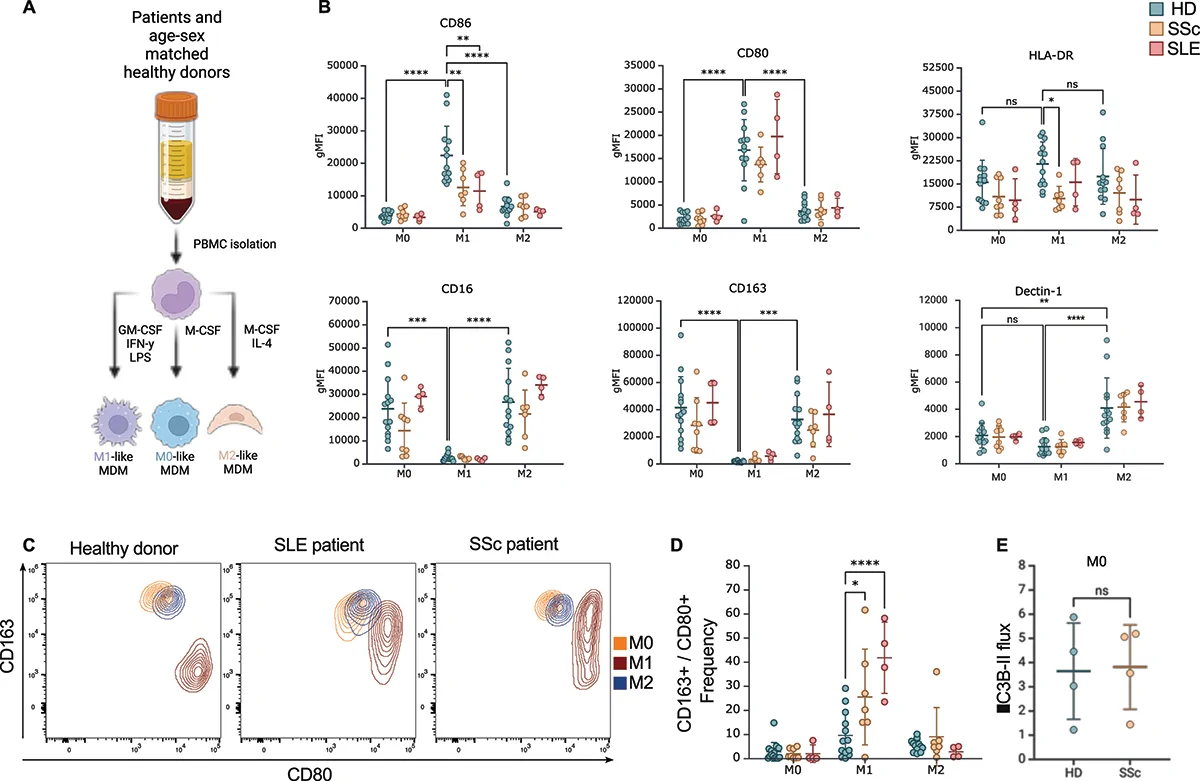
M1, but not M0 or M2, macrophage differentiation is altered in SSc patients compared to healthy donors. (A) Diagram summarizing the *ex vivo* experimental procedures for differentiating CD14-positive monocytes into M0-, M1-, or M2-polarized monocyte-derived macrophages (MDM). (B) Surface expression levels (mean fluorescence intensity) of macrophage polarization markers (CD86, CD80, HLA-DR, CD16, CD163, and Dectin-1) analyzed by flow cytometry. (C) Representative dot plots illustrating the CD163/CD80 double-positive macrophage populations under M0, M1, or M2 skewing conditions in healthy donors (blue), SLE patients (red), and SSc patients (yellow). (D) Corresponding quantification of the CD163/CD80 double-positive cell proportions. (E) Quantification of basal autophagic flux (LC3B-II flux) in M0 MDM by flow cytometry. Statistical analyses were performed using two-way ANOVA with Tukey’s multiple comparisons test (B, D) or a two-tailed Wilcoxon test (E). *p < 0.05, *p* < 0.005, ***p < 0.001, ****p < 0.0001; ns = not significant.

### Evaluation of LAP activity of M0 monocyte-derived macrophages (MDM) from SSc and SLE patients

As the differentiation yield of M0 cells and autophagy levels are comparable between SSc patients and HD patients, we investigated whether LAP, which is active in professional phagocytes, was impaired. To evaluate LAP activity, we incubated macrophages with an LAP-inducing stimulus (IgG-coated beads) and non-coated beads which should only induce phagocytosis (Figure 3(A)). We first showed the specific induction of LAP with IgG-coated beads compared with non-coated beads that only induced phagocytosis (Figure 3(B) and figure S2). We then compared the levels of phagosomes per cell in SSc macrophages between patients and healthy subjects. We found no significant differences between the SSc and HD groups (Figure 3(C)). Interestingly, the SLE samples tested were in the lower range, consistent with phagocytic defects characteristic of the pathology. To measure LAP activity, we stained the samples with anti-LC3 antibody, allowing the discrimination of phagosomes from LAPosomes (LC3-II-positive phagosomes, Figure 3(B) and S2). We observed a reproducible decrease in the number of LAPosomes in SSc macrophages compared to that in their matched controls, while the number of total phagosomes is comparable (Figure 3(C, D)). This finding was confirmed by calculating the ratio of LAPosomes to total phagocytic vesicles, which demonstrated a marked reduction in LAP activation (Figure 3(E)). In contrast, the SLE samples tested were in a higher range of LAPosome frequency found in SSc, but still in the lower range compared with HD.

**Figure 3.**
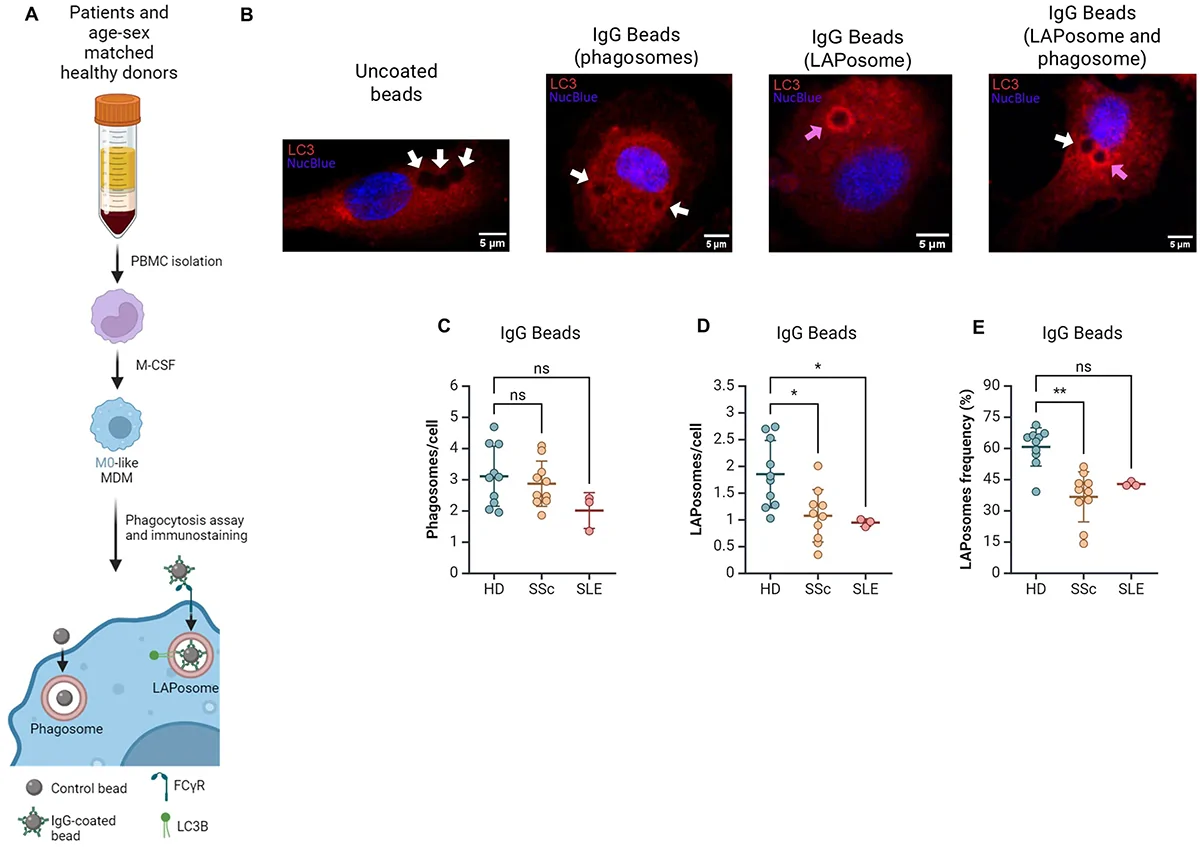
LC3-associated phagocytosis is defective in M0 MDM from SSc patients. (A) Schematic overview of the LAP induction assay using IgG-coated beads versus bovine serum albumin (BSA)-coated control beads. (B) Representative immunofluorescence images of MDM following phagocytosis. White arrows indicate conventional phagosomes, while magenta arrows denote LAPosomes (LC3-positive phagosomes). Higher magnification insets are provided to clearly distinguish LC3-positive vesicular structures. Quantification of the mean number of (C) phagosomes per cell and (D) LAPosomes per cell following the uptake of IgG-coated beads. (E) Overall LAP activity expressed as the frequency of LAPosomes relative to total phagosomes. Each data point represents an independent experiment with at least 45 cells analyzed per sample (Healthy donors: *n* = 10; SSc patients: *n* = 10; SLE patients: *n* = 3). Statistical analyses were performed using the Kruskal–Wallis test with Dunn’s multiple comparisons test.

## Discussion

This is the first study assessing autophagic activity in PBMC from patients with SSc and to report that no major defects in canonical autophagy were detected in circulating immune cells. We observed a decrease in autophagic flux in T cells after stimulation. This is reminiscent of what has been found in SLE T cells in several reports showing abnormal accumulation of autophagic vesicles [21] or LC3-positive compartments [22,23]. The increase in LC3 staining associated with reduced LC3-II accumulation after lysosomal degradation blockade suggests an impaired autophagic process in stimulated SSc T cells, which could be linked to an imbalance between autophagosome formation and degradative capacity. Further studies using additional autophagy markers, such as p62 will be required to confirm a specific alteration of the canonical autophagy pathways. Such approaches could determine whether the defect occurs at the degradation step of the autophagic process after stimulation in SSc T cells. It will then be relevant to define the consequences of this deregulation in the T-cell polarization profile and survival. Several mouse models have shown that inhibiting autophagy in lymphocytes leads to the amelioration of lupus disease [24–26]. Whether similar mechanisms contribute to SSc pathogenesis remains to be determined.

We then focused on the role of autophagy machinery in differentiated macrophages. We found no difference in autophagic activity between macrophages from patients with SSc and those from healthy subjects. It has been reported that inhibition of selective forms of autophagy in phagocytes can worsen disease severity [27]. Further investigation of specific autophagy pathways, such as mitophagy, in SSc macrophages could be performed to identify possible defects that may not have been detected in our study.

We reported as others that SSc monocyte-derived macrophages are biased toward atypical mixed M1/M2 phenotypes under certain conditions [20]. These intrinsic differences in macrophage polarization may be linked to impaired efferocytosis. Such clearance defects are central to several autoimmune pathologies, such as SLE, and have been more recently described in SSc. Efferocytic defects in SSc have been proposed to contribute to the accumulation of cell debris and subsequent inflammation and autoantigen presentation, as observed in SLE [6,7]. This could lead to both disease progression and the IFN signatures more recently described in patients with SSc [28]. We did not evaluate efferocytic activity using M1 and M2 differentiation protocols. This investigation would be of interest considering the mixed M1/M2 phenotype observed in macrophages differentiated from SLE and SSc.

We then compared populations with the same polarization profiles for efferocytosis and LAP. We chose to focus on M0 differentiation protocols because this setup resulted in minimal phenotypic differences between healthy controls, SSc, and SLE samples. Although the use of M-CSF may induce macrophage polarization, we obtained a distinct phenotype from that of M2 macrophages particularly based on Dectin-1 expression.

The most striking result we report here is the intrinsic impairment of LAP in SSc M0 macrophages. We are confident that our assay indeed measures LAP activity as no LC3 decoration of phagosomes was observed with non-IgG-coated beads. Although FcγR engagement is a well-known activator of LAP, it would be interesting to delineate the precise CASM mechanism involved using specific inhibitors targeting NOX2, RUBICON or vATPases. LAP contributes to efferocytosis and is involved in limiting IFN signaling after engulfment of dead cells [14]. We hypothesize that LAP defects in SSc could therefore contribute to enhanced inflammation and the type I IFN signature observed in patients with SSc. Functional assays linking LAP efficiency, type I interferon and inflammatory mediator release will be required to determine the potential physiological relevance of LAP defects in SSc.

We found no correlation between LAP activity, clinical score, treatment, or organ involvement (not shown). This could be explained by the relative homogeneity of our cohort. Of note, this study is limited do diffuse SSc and our findings cannot be extrapolated to limited cutaneous SSc or earlier preclinical stage. Eight patients out of 10 studied for LAP could be considered as having early disease (all had diffuse SSc, with disease assessment performed less than 5 years after the first symptom, excluding Raynaud Syndrome). It could be relevant to study LAP activity in less severe SSc to assess whether its impairment is as pronounced as that observed in our cases. Moreover, we only tested LAP defects in our M0-differentiation conditions, as this setup allowed a comparable efficiency and phenotype between healthy controls, SSc, and SLE samples. We cannot exclude that M-CSF treatment used for this polarization protocol may influence macrophage phagocytic properties. It would therefore be interesting to measure LAP activity in macrophages differentiated without exogenous M-CSF. Additionally, isolating circulating monocytes from SSc patients exhibiting a mixed M1/M2 phenotype [4,29] by sorting followed by LAP assessment could provide insight into the intrinsic properties of patient-derived macrophages without the potential bias introduced by *in-vitro* differentiation protocols. Although these results obtained in M0 macrophages are interesting, they should be complemented by studies using spontaneous or alternative differentiation protocols to further understand the mechanisms underlying the observed regulation.

We also reported a tendency for defective efferocytosis and LAP in SLE macrophages (with decreased number of phagosomes per cell and reduced LAPosomes frequencies, similarly to SSc macrophages). Although this defect appeared more pronounced in SSc, this observation remains exploratory given the modest number of SLE samples assessed.

There may be multiple consequences of LAP defects in SSc. Interestingly, LAP has been shown to be induced in liver fibrosis and to exert a protective role against inflammation [30]. Inhibition of LAP exacerbates the inflammatory signature of human monocytes from cirrhotic patients and in mouse models of chronic liver injury. LAP is proposed to be induced in this context by the engagement of immune complexes by FcγRIIA, leading to inhibitory ITAM (ITAMi) signaling and recruitment of the phosphatase SHP-1, which may explain the anti-inflammatory effects. Interestingly, CD16 expression was linked to the M2 phenotype, which is considered to be the most efficient phenotype for efferocytosis. Thus, we speculated that LAP activity may be increased in M2 macrophages. In M0 cells, in which we assessed LAP activity, no significant difference was found in CD16 surface expression between SSc patients and HD (not shown). Moreover, no correlation was found between CD16 expression levels and LAP activity in SSc M0 cells, which could be due to the relatively small cohort. It would be relevant to investigate FcγR downstream signaling, such as SYK recruitment, ITAMi activation, and its association with SHP-1, to identify the molecular causes of LAP deregulation in SSc. In any case, future studies should focus on identifying the mechanisms underlying LAP deregulation in M0 macrophages from SSc patients. One possibility could involve altered CASM regulation (e.g. through the ATG16L1/V-ATPase activity [31] or responses to STING activation which is aberrantly regulated in SSc [32]).

Our study points toward a possible pathological mechanism involving to LAP activity in macrophages. Therefore, it will be important to evaluate the impact of LAP modulation on the inflammatory potential of SSc macrophages. Specific LAP modulators can be considered as potential therapeutic approaches [11,33]. It was recently shown that columbamine enhances macrophage-mediated efferocytosis by promoting LAP and protecting against intestinal inflammation in a murine colitis model [34]. LAP activity may therefore attenuate fibrosis in SSc, as previously proposed. We propose that enhancing LAP activity could also contribute to limiting autoimmune and inflammatory responses.

## Supplementary Material

Supplemental Material

Supplemental Material

## Data Availability

All data relevant to the study are included in the article or are uploaded as supplementary information. A preprint version of this manuscript is accessible [35].
